# The Relationship between Happiness and Mental Health among Saudi Women

**DOI:** 10.3390/brainsci13040526

**Published:** 2023-03-23

**Authors:** Noura Abdulla Almadani, Mai B. Alwesmi

**Affiliations:** 1 Community Health Nursing Department, College of Nursing, Princess Nourah bint Abdulrahman University, P.O. Box 84428, Riyadh 11671, Saudi Arabia; naalmadani@pnu.edu.sa; 2 Medical-Surgical Nursing Department, College of Nursing, Princess Nourah bint Abdulrahman University, P.O. Box 84428, Riyadh 11671, Saudi Arabia

**Keywords:** happiness, mental health, stress, depression, anxiety, insomnia, social dysfunction

## Abstract

The happiness and mental health of individuals are crucial for national developments. In Saudi Arabia, wellbeing occupies a central position in Vision 2030, along with women’s empowerment. Rapidly changing rights and responsibilities might result in more sources of stress. The aim of this study was to explore happiness and mental health among Saudi women during their contributions to a fast-growing nation in all fields. We assessed happiness and mental health (somatic symptoms, depression, anxiety, insomnia, and social dysfunction) in 308 Saudi women aged 15–50 years using a self-administered online survey comprising the short Oxford Happiness Questionnaire (OHQ-8) and the General Health Questionnaire (GHQ-28). The participants demonstrated general satisfaction yet showed rising psychological distress. Married women reported better mental health compared to single women, particularly with regards to anxiety, insomnia, and depression. Depression was highest among younger women. Employed women demonstrated lower social functioning compared to unemployed women. Women with a higher educational level showed lower social functioning. Happiness scores were significantly and inversely related with overall mental health scores as well as mental health subscales (somatic, anxiety, and depression), except for social dysfunction, which showed a positive correlation to happiness scores. This study contributes to the body of literature on women’s mental health and happiness by providing recommendations for improving both as well as directions for future research.

## 1. Introduction

Happiness, defined in terms of “frequent positive emotions and high life satisfaction” [1] (p. 2), is a complex but highly valued construct at the confluence of the individual and the contextual. Happiness involves income, employment, and basic needs, as well as marital status, familial and social relations, senses of equality and inequality, and physical and mental health [2]. Happiness is distinct from, but reciprocally related to, social and economic wellbeing, and both are situated within health, which the World Health Organization defines as “a state of complete physical, mental, and social well-being and not merely the absence of disease or infirmity” [3]. Mental health and happiness are key factors for productivity at the workplace [4,5], and many countries have identified happiness among citizens as of primary importance, far beyond the gross domestic product. For example, quality of life measures include the OECD Better Life Index and the Canadian Index of Wellbeing. In Saudi Arabia, happiness is central to the recent launch of the reform initiative, Vision 2030, which aims for economic transformation, enhanced community health, and increased social wellbeing [6,7]. Saudi Arabian women occupy a central position in Vision 2030, and its initiatives aim to enhance the status of women and achieve gender equality through securing women’s rights, empowering women at community, national, and international levels, and supporting women’s participation in the economic development of the country. Currently, Saudi women above 15 years of age represent 49% of the population, with an average age of 28 years; Saudi women above 15 years of age and below 65 years of age represent 32.4% of the Saudi workforce [8]. The government of Saudi Arabia is making efforts to increase this figure of women in the workforce by promoting women’s engagement in economic development with a focus on gender equality [7]. With Vision 2030, the Saudi government has promised: “With over 50 percent of our university graduates being female, we will continue to develop their talents, invest in their productive capabilities, and enable them to strengthen their future and contribute to the development of our society and economy”. Individual happiness has been recognized as an important aspect of social and national wellbeing, and links have been made visible between individual happiness, physical and social health, and national development; however, happiness among women specifically in Arab-Muslim contexts requires more research.

The research on the subject of mental health and happiness has a long history, with various definitions and theoretical perspectives. Mental health refers to the state of well-being in which an individual can realize their potential, cope with everyday stressors, and contribute to their community [4]. Happiness, on the other hand, is a subjective feeling of positive emotions and satisfaction with one’s life. One of the fundamental theoretical perspectives that underpins the study is the positive psychology approach, which focuses on studying the factors that contribute to positive emotions, behaviors, and experiences [5,6]. This approach has been applied to various fields, including mental health, social psychology, and organizational behavior.

In striving to determine what makes a person well, the term wellbeing is often used interchangeably with other terms such as “happiness”, “flourishing”, “enjoying a good life,” and “life satisfaction” [9] (p. 20), as collective aspects of wellbeing, each of which has different intrinsic meanings, pathways to expression, and methods of empirical investigation. Happiness is often described in terms of subjective wellbeing, as hedonic focus on wellbeing as positive and negative affect, happiness, and quality of life satisfaction [10]. Another orientation is psychological wellbeing, which takes an eudaimonic focus on life meaning, purpose, and the perceived contributions of an individual in relation to society as “characteristics of what it means to be mentally healthy, fully developed, purposefully engaged, self-actualized, fully functioning, and mature” [11] (p. 242). Psychological well-being has been associated with physical health and lower incidences of chronic diseases [12]. Taken together, both subjective and psychological wellbeing are aspects of happiness that constitute a robust focus on positive aspects of psychology, which tend away from the study of disorder, illness, and pathology towards “the study of the conditions and processes that contribute to the flourishing or optimal functioning of people, groups, and institutions” [13] (p. 104).

This study is concerned with happiness, defined as “frequent positive emotions and high life satisfaction” [1] (p. 2), which is currently central to research on positive psychology and associated with greater physical health by promoting healthy lifestyles and helping people deal with stress [12], financial wellbeing [14], and stronger human relationships [15], including marital relationships. These factors linking happiness with other health indicators make happiness a goal for nations aiming to increase the wellbeing and overall health in the general population. Taking a broad view on happiness and wellbeing, Saudi Arabia’s Vision 2030′s strategic objectives include improving healthcare services access and value, reducing health threats, promoting sports activities across society, enhancing service quality, decreasing pollution, and using social systems to empower people, especially women [7].

More broadly, research on women’s happiness and wellbeing within a Saudi and Muslim context is a unique subset of the literature on happiness. Several studies were conducted before Vision 2030 that examined happiness in Saudi, including Aboalshamat et al. (2018), who focused on resilience and happiness among dental and medical students in Jeddah and reported a correlation between the two: students with increased resilience were happier and more satisfied with their lives, but this correlation was higher among female students than male ones [16]. Jradi and Abouabbas (2017) conducted a cross-sectional survey of women in Riyadh and found that women experiencing poor physical conditions and violence had impoverished senses of well-being [17]. Alhakami and Baker (2018) examined happiness among nurses and found a correlation between happiness and awareness of the influence of their work [18]. Esmail and Shili (2018) gathered questionnaire data to examine the relationship between happiness, social factors, and economic development among people in Jazan, Saudi Arabia, and found that although happiness contributes to economic development via increased motivation, they identified social factors as a main source of happiness, including health care, education, employment, and marital status [14]. In a review of the literature on social media data as a source of sentiment analysis research, Alharbi, Alotebii, and AlMansour (2018) proposed using Twitter posts to measure happiness levels in different places and identify activities or circumstances that contribute to people’s happiness [19].

According to the General Authority for Statistics in the Kingdom of Saudi Arabia, a majority of Saudi working women have reported good self-rated health [8]. However, few studies have explored the impact of happiness on women’s health since the launch of Vision 2030 in 2016. Increasing happiness among Saudi women has been reported, including originating in women having the right to drive [20], along with increasing professional opportunities. Alquwez et al. (2021) conducted a validation study on the psychometric properties of the Subjective Happiness Scale in Arabic (SHS-A) among 300 Saudi working women. Along with finding evidence that supports the validity and reliability of the SHS-A with working Saudi women, they also found that employment position, working hours, and monthly salary influenced the subjective happiness of the women [21]. Moreso, recently, in a study of self-esteem with 1883 Saudi women aged 30–75 years, Kazi (2021) found that emotional and tangible support showed the strongest positive correlation with self-esteem levels, followed by education and physical education. The study suggests the implementation of social support and counseling programs, especially for socially isolated women who have low self-esteem [15].

In short, numerous factors affect women’s physical and mental health, including happiness, generally and among Saudi women specifically, such factors include social support, education, physical activities, and access to healthcare facilities [15,16]. Building upon the few previous studies exploring subjective happiness with working Saudi women [8], sources of mental health and wellbeing among Saudi women (e.g., Aboalshamat et al., 2018; Jradi and Abouabbas, 2017) [16,17], and responding to calls for more research on Saudi women’s happiness, this exploratory study set out to examine happiness levels among Saudi women as related to mental health.

The study draws on previous research that has established a link between mental health and happiness. For instance, studies have shown that individuals who experience psychological distress are more likely to report low levels of happiness and life satisfaction [8,16,17]. Other studies have found that interventions aimed at promoting positive emotions and well-being can have positive effects on mental health outcomes. The study is also closely related to research that has investigated the role of cultural factors in shaping mental health and happiness. Given that the study focuses on Saudi women, it is important to consider the cultural context in which these women live, including the social norms, values, and beliefs that may impact their mental health and happiness.

The three aims of the study were firstly to explore happiness among Saudi women in a Vision 2030 sociocultural climate, secondly to screen for mental health and potential psychiatric disorders, and thirdly, to examine the relationship between the two. As such, this study contributes to evaluating the quality of life expressed in health and happiness among women as a key goal of Vision 2030. By shedding light on the intersection of mental health and happiness in the context of Saudi Arabia’s Vision 2030 initiative, this study breaks new ground in our understanding of the complex factors that influence women’s well-being in rapidly changing sociocultural environments.

## 2. Materials and Methods

The study aimed to explore mental health and happiness among Saudi women in Riyadh using an online survey with convenience sampling during June 2021. The questionnaire used in the study was a unidimensional scale questionnaire that adapted two validated instruments: the short Oxford Happiness Questionnaire (OHQ-8) and the General Health Questionnaire (GHQ-28). The OHQ-8 was developed by Hills and Argule in 2002 [22] and measures happiness levels, whereas the GHQ-28 was developed by Goldberg and Hillier in 1979 [23] and assesses general mental health. The questionnaire also included questions about sociodemographic characteristics, such as age, employment, educational level, and marital status. After obtaining ethical approval, the questionnaire was posted on Google Docs and disseminated through WhatsApp. The study’s sampling technique was convenience sampling, which is a non-probability sampling technique that selects participants based on their accessibility and willingness to participate. To the best of our knowledge, there have been few, if any, studies that have employed the OHQ-8 and the GHQ-28 together in Arabic. However, some studies have used these instruments separately in the Arabic language.

The study aimed to investigate the sociodemographic characteristics of individuals belonging to different age groups, ranging from 15 to 50 years old. To achieve this objective, the study compared individuals with varying educational backgrounds, including those with school, bachelor’s, master’s, and PhD degrees. Moreover, the study assessed differences between individuals who are single, married, divorced, or widowed and their employability status, which may impact their ability to access certain resources or participate in certain activities. The variables under investigation included a range of sociodemographic factors, such as income, occupation, education level, and marital status. By examining these variables in relation to the characteristics of different age groups and educational backgrounds, the study aimed to identify any significant differences or trends that could inform policy or practice in relevant fields.

The study used convenience sampling to select participants based on accessibility and willingness to participate. The inclusion criteria were Saudi women aged 15 or older who could read Arabic and provided informed consent. Those with diagnosed mental health disorders or receiving treatment were excluded, which may limit the generalizability of the findings.

To protect the anonymity of participants, a unique identifier was assigned to each individual instead of using personal information. Confidentiality was maintained by safeguarding the data and restricting access to authorized personnel. Informed consent was obtained by informing participants of the study’s purpose, potential risks and benefits, and how their information would be utilized and safeguarded. Participants voluntarily provided their informed consent to take part in the study. The data was only accessible to those involved in the study and was securely deleted when no longer necessary.

### 2.1. The Short Oxford Happiness Questionnaire (OHQ-8)

The short Oxford Happiness Questionnaire (OHQ-8) is a shorter version of the OHQ-29 that was designed by Hills and Argule (2002) to examine personal happiness and subjective wellbeing [22]. The original OHQ-29 followed the format of the Beck Depression Inventory [24], whose 20 items were reversed, and nine additional items were added to cover aspects of happiness. OHQ-29 is a single-item questionnaire that can be answered on a uniform six-point Likert scale. Almost half of the items (14) are negatively formulated to reduce respondent bias. OHQ-8 was extracted based on factorial analyses of OHQ-29, and its eight items have been deemed sufficient to discriminate respondents’ scores with an accuracy of 90%. The instrument has been reported to have a retest reliability of 0.91 and an inter-item correlation of 0.04–0.65 with a mean score of 0.28 [22].

Validated in several languages, the OHQ-29 is a popular and reliable instrument. Several studies have used and validated the Arabic version of this instrument, including Alansari and AlAli (2017), who evaluated the psychometric properties of the Arabic adaptation of the OHQ-29 and found satisfactory internal consistency and overall validation. They recommended it as a measure of happiness in Arabic-speaking populations [25]. Abdel-Khalek (2013) used the instrument in measuring the construct validity of an Arabic Scale of Happiness, which he designed for use in an Arabic-speaking Kuwaiti context. In computing the Pearson correlation coefficient between two groups who responded to the two scales, good construct validity was found between them [26]. In another study exploring associations between religiosity, generalized self-efficacy, mental health, and happiness among Arabic-speaking students in Kuwait, Abdel-Khalek and Lester (2017) computed the criterion-related validity for their rating scale of happiness against the Oxford Happiness Inventory (Argyle et al., 1995), previously translated into Arabic by Abdel-Khalek, and high levels of reliability and validity coefficient were reported [27]. In a later study, Dodeen and Cody (2021) conducted statistical analyses of this Arabic Scale of Happiness as correlated to the OHQ and found it to be a reliable and valid scale for measuring happiness among Arab college students [28]. In Saudi Arabia, specifically, Torchyan et al. (2016) used the OHQ-8 to examine happiness and physical activity levels among Saudi male and female undergraduate medical students in Riyadh. They reported an interaction between physical activity and happiness with male students, whereby male students with both low levels of happiness combined with low levels of physical activity constituted the group most likely to initiate smoking [29].

### 2.2. The General Health Questionnaire (GHQ-28)

A second instrument, the General Health Questionnaire (GHQ-28), is a unidimensional scale designed as a screening tool for mental health disorders, whereby higher scores indicate a higher level of stress. This questionnaire includes four subscales that assess somatic symptoms (depression, anxiety, and insomnia) and social dysfunction [23]. The GHQ dichotomous scoring method (0-0-1-1) was applied, assigning a score of 0 for “not at all”, “no more than usual” or their equivalence, and a score of 1 for “rather more than usual”, “much more than usual,” or their equivalence. According to Goldberg and Hillier (1979), the best overall cut-off level was 4/5, distinguishing between normal individuals and subclinical or clinical cases with a sensitivity of 87% and specificity of 75% [23]. This score was validated for an Arabic version in Saudi Arabia by Alhamad and Al-Faris (1998), employed to improve detection of psychiatric morbidity and in epidemiological research [30]. Another study was conducted to estimate the psychometric properties of the Arabic GHQ-28 against the Beck Depression Inventory (BDI-II) for screening depression in war-exposed civilians [31]. Two data sets collected in a civilian sample from south Lebanon were analyzed. Internal consistency in the two samples was high for the GHQ-28 (0.91 and 0.80) and the BDI-II (0.88 and 0.84). The BDI–II was significantly correlated with the GHQ-28 total score and the Depression subscale. The Arabic GHQ-28 was found to be a valid instrument for screening for depression in the studied population [31]. Finally, El-Metwally et al. (2018) set out to assess the factorial structure of the GHQ-12 in a mixed-gender population in a central Saudi Arabian region. They found that the instrument showed strong psychometric characteristics and recommended it as a reliable and valid instrument for recognizing people who are at a greater risk of suffering from mental health issues and in order to optimize health [32].

### 2.3. Content Validity and Ethical Approval

To test their content validity, the translated instruments were submitted to three experts in community health. Cronbach’s alpha was used to test the internal consistency of the items to test reliability for both the OHQ-8 and the GHQ-28. The survey was preliminary and conducted on five random participants to test clarity, applicability of the instruments, and feasibility of the research application.

Ethical approval was obtained from the first author’s university, protocol code 21-0269, date of approval 6 June 2021, and ethical considerations were followed throughout the study process, including inviting participants on a voluntary basis and reminding them that they could terminate participation at any time. The data were anonymized, kept confidential, and encrypted on the first author’s computer.

### 2.4. Strategies to Address Limitations of Self-Administered Online Data Collection in Research

Self-administered online data collection is convenient and cost-effective, but there are several limitations to be aware of. These include self-selection bias, limited control over the research environment, response validity issues, technical difficulties, and a lack of personal interaction. These limitations can impact data validity and reliability, and researchers should take steps to address them when using this method of data collection.

To mitigate the limitations of self-administered online data collection, researchers can take steps such as ensuring transparency in recruitment, using quality assurance measures, including validation measures, and interpreting results in light of the limitations. By doing so, researchers can increase the validity and reliability of the data obtained and ensure that the results are interpreted accurately.

### 2.5. Statistical Analysis

Data analyses were performed using IBM^®^ SPSS^®^ Statistics software version 28.0. Cronbach’s alpha estimates reliability at 0.89 for GHQ-28 and at 0.75 for OHQ-8. The normality of the data set was assessed by the Shapiro–Wilk test. The equality of variances between the groups was assessed with Levene’s test. Demographic comparisons were made by Mann–Whitney U tests for employability groups and Kruskal–Wallis H tests followed by a Dunn–Bonferroni post hoc test for other demographic groups. All correlations were assessed by Spearman’s rank correlation coefficient.

## 3. Results

### 3.1. Demographic Characteristics Overview

A total of 308 Saudi women completed the survey, their demographic characteristics are summarized in Table 1. A majority of them were within the age group 21–30 (*n* = 95). Followed by 15–20, 31–40, and ˃40, respectively. Most of the women participated in the study were married (*n* = 194, 63%) with a minority widowed (*n* = 10, 3.2%). Their educational level was mostly a bachelor’s degree (*n* = 203, 65.9%) with minority of PhD holders (*n* = 25, 7.2%). Just over half of them were employed (*n* = 179, 58.1%). 

### 3.2. Oxford Happiness Questionnaire

Overall OHQ–8 results showed general satisfaction among the population with overall score of 4.19 ± 0.56. Of 308 respondents, 60.1% reported their happiness level as (4.13–5.50), 39.3% were not particularly happy or unhappy, and 0.6% were not happy (Table 2). 

### 3.3. General Health Questionnaire

Participants demonstrated an overall GHQ score of 11.15 ± 6.60 (Table 3). The majority of them (82.5%) had a GHQ score of 6 to 28. Among the four GHQ categories, social dysfunction (4.01 ± 2.38) represented the greatest contribution to the total score. To explore relationship between happiness and mental health, participants were grouped according to their mental health scores into healthy group; scored ≤5, the best reported cut-off point that distinguished between normal individuals and subclinical/clinical cases [23,30], individuals scored ˃5 were then divided into subgroups according to quartiles Q1 = 8, Q2 = 12, Q3 = 17 and Q4 = 28, indicating mental health disorder severity, giving a total of five subgroups: 0–5 (*n* = 54, 17.5%), 6–8 (*n* = 66, 21.4%), 9–12 (*n* = 67, 21.8%), 13–17 (*n* = 63, 20.5%), 18–28 (*n* = 58, 18.8%). The differences between these groups on OHQ score were then compared.

### 3.4. Demographic Comparisons

#### 3.4.1. Age Group

**Figure 1:**
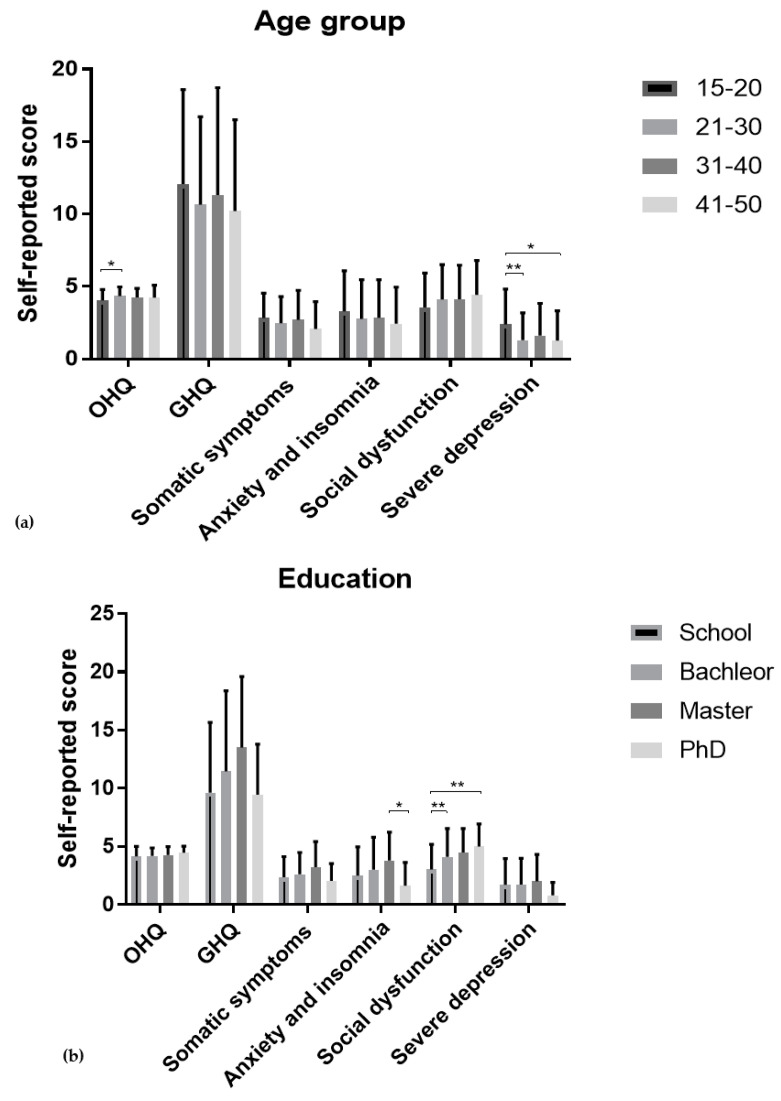

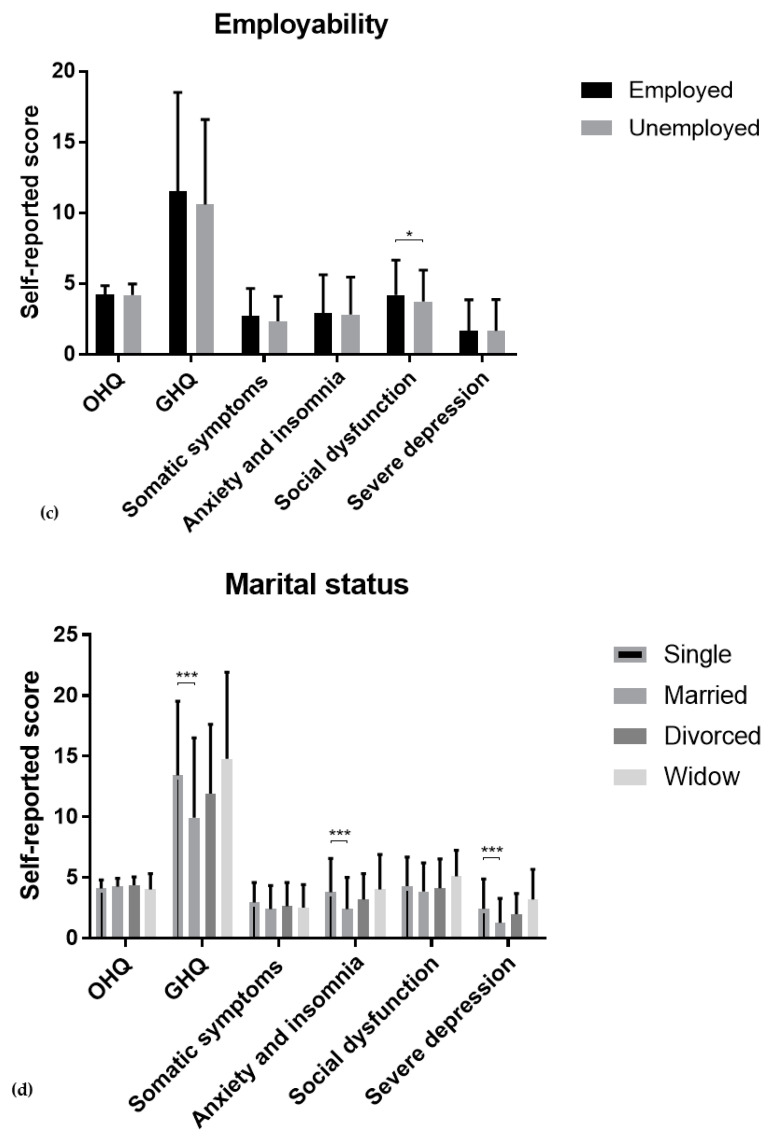
Bar charts for (**a**) Age group, (**b**) Education, (**c**) Employability and (**d**) Marital status represent Mean ± *SD*. * *p* < 0.05, ** *p* < 0.005, *** *p* < 0.001.

The Kruskal–Wallis H test showed that there was a statistically significant difference in OHQ score between the different age groups, χ^2^(3) = 8.253, *p* = 0.041. The age group 21–29 were significantly slightly happier than 15 -20, *p* = 0.040, Dunn–Bonferroni post hoc test. In GHQ, only the depression score showed a significant difference among age groups, χ^2^(3) = 14.014, *p* = 0.003. The age group (15–20) was significantly more depressed compared to (21–30) and (41–50) age groups *p* = 0.008 and *p* = 0.010, respectively, Dunn–Bonferroni post hoc test (see Figure 1).

#### 3.4.2. Marital Status

The results of the Kruskal–Wallis H test revealed that there was no difference between marital status groups in happiness score; however, it showed a significant difference in mental health score χ^2^(3) = 24.348, *p* < 0.001. Married participants had a significantly better mental health than single participants, *p* <0.001, primarily in anxiety and insomnia and severe depression, χ^2^(3) = 16.083, *p* = 0.001 and χ^2^(3) = 23.210, *p* < 0.001, respectively. Single participants showed significantly poorer anxiety and insomnia and severe depression scores compared to married participants, *p* ≤ 0.001, Dunn–Bonferroni post hoc test (Figure 1). 

#### 3.4.3. Employment

The Mann–Whitney U test results showed no difference between employed and unemployed individuals in happiness and overall mental health scores. Group comparison on mental health subscales revealed that unemployed individuals had a slightly better social function than employed individuals U = 9999.0, *p* = 0.043 (Figure 1).

#### 3.4.4. Education

The Kruskal–Wallis H test results showed no difference between individuals with different educational levels in happiness and overall mental health scores. Mental health subscales anxiety and insomnia and social dysfunction showed difference between the groups, χ^2^(3) = 10.134, *p* = 0.017 and χ^2^(3) = 16.483, *p* < 0.001, respectively. PhD holders showed a better anxiety and insomnia score than MA holders (*p* = 0.018). Individuals with school degree showed better social function compared to bachelor and PhD holders; *p* = 0.007 and *p* = 0.002, respectively (Figure 1). 

### 3.5. Happiness Scores as Related to Mental Health

Spearman’s rank correlation coefficient showed that happiness score significantly negatively correlated with overall mental health scores, as well as mental health subscales, somatic, anxiety, depression, *r* = −0.266, −0.284, −0.345 and −0.393, respectively, *p* < 0.001, except of social dysfunction which showed positive correlation to happiness score, r = 0.274, *p* < 0.001 (Table 4).

### 3.6. Happiness Comparisons between Mental Health Subgroups

**Figure 2:**
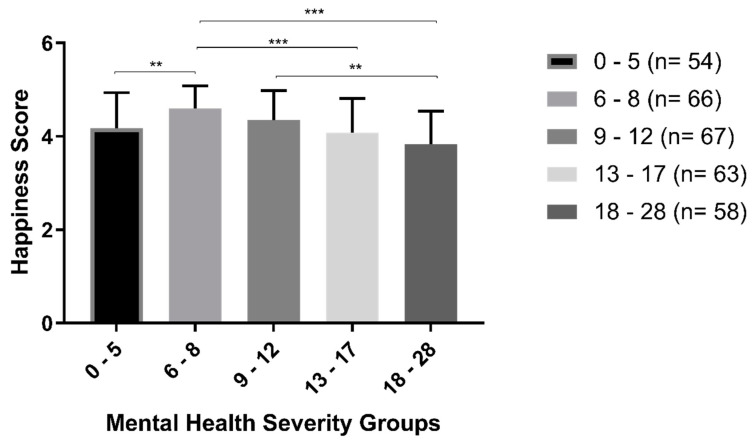
Mental health severity groups comparison on happiness level. The bars represent Mean ± *SD*. ** *p* < 0.005, *** *p* < 0.001.

The Kruskal–Wallis H test showed that there was a statistically significant difference in OHQ score between mental health subgroups, χ^2^(4) = 42,529, *p* < 0.001, with a main rank happiness level of 149.14 for 0–5, 204.25 for 6–8, 168.57 for 9–12, 137.03 for 13–17, and 105.59 for 18–28. Figure 2 displays a bar graph illustrating the comparison of mental health severity groups on happiness levels. The mental health severity groups were divided into four categories: healthy (0–5), mild (6–8), moderate (9–12), and severe (13–28). The *y*-axis represents happiness levels, and the *x*-axis represents the mental health severity groups. The bars in the graph represent the mean happiness levels for each group, along with the standard deviation (*SD*).

The graph shows that there is a slight but significant decrease in happiness levels as the mental health disorder score increases. Specifically, the healthy group (0–5) showed a significantly lower happiness level than the mild group (6–8). The mild group (6–8) was significantly happier than the moderate group (13–17) and the severe group (18–28). The moderate group (9–12) was significantly happier than the severe group (18–28).

The statistical significance of the differences in happiness levels between the groups was indicated by asterisks. The significance level was set at *p* < 0.005 for double asterisks (**) and *p* < 0.001 for triple asterisks (***). Overall, the results suggest that mental health severity is inversely related to happiness levels, with individuals with greater mental health disorder scores reporting lower levels of happiness.

## 4. Discussion

The current study aimed to assess the relationship between happiness and mental health among Saudi women. The findings suggest that the majority of these women are generally happy and mentally healthy, and that happiness and the potential to develop psychological disorders are generally negatively correlated. A demographic finding that singles showed poorer mental health compared to married, particularly in anxiety, insomnia, and depression, echoes previous research suggesting that married people have better mental health over the long-term (e.g., Yap and Lucas, 2012) [33]. The group of 15–20-year-old Saudi women showed the greatest depression score compared to the older groups; they were also less happy than 21–30-year-old women.

As an exploratory study, it raises some questions that point to directions for further research. For example, when categorizing the population according to their mental health severity based on GHQ score, the group below the threshold score, considered to be the healthiest, showed lower happiness score compared to a group with poor mental health. However, they were expected to show the highest happiness score. 

In addition, in the subscales of GHQ, social dysfunction showed the greatest contribution to the overall score. In other words, the happiness score significantly negatively correlated with overall mental health scores, as well as mental health subscales of somatic, anxiety, depression, except for social dysfunction, which showed a positive correlation to happiness. A question arises: was the language used in social dysfunction questions clear enough to the participants? Prior research indicates that Saudi women in this population are engaged on social media [34], albeit to a lesser extent than men [35]. This suggests that the questionnaire, which was originally intended for face-to-face interaction prior to the emergence of social media, may be identifying social dysfunction in an obsolete manner. Further research is required to probe this hypothesis and to examine in more detail the functional significance of this finding. 

Although the population showed an overall score of general satisfaction in OHQ, their self-rated score from GHQ showed a tendency to develop mental health disorders. This finding raises another question: as previous studies reported employing this instrument did so with a diagnosed population, are these tools accurate in assessing happiness and mental health in a general population?

The findings of the study highlight several noteworthy differences in various measures of mental health and well-being across diverse demographic groups. Firstly, there is a significant difference in both happiness levels and the severity of depression scores between the age groups of 15–20 and 31–30, as well as between 15–20 and 41–50 on the severe depression score. This finding is consistent with Allahverdipour et al. [36]. Furthermore, educational levels were compared, and it was discovered that there is a significant difference in anxiety and insomnia scores between individuals with a master’s degree and those with a PhD. Additionally, a significant difference was observed in social dysfunction between individuals with school-level education and those with a master’s degree or PhD. These results align with Cetin et al. [37]. Moreover, a comparison between employed and unemployed women revealed a significant difference in social dysfunction. Lastly, marital status was compared, and it was observed that there is a significant difference in the GHQ overall score, anxiety and insomnia score, and severe depression score between singles and married women. This result is in line with the research conducted by Khumalo et al. [38] and Huppert [39].

These results suggest that various demographic factors can play a role in mental health and well-being and may need to be taken into consideration when developing interventions or providing support. The study categorized the mental health severity of participants into four distinct groups based on their scores, which were healthy (0–5), mild (6–8), moderate (9–12), and severe (13–28). The findings of the study reveal a noteworthy yet statistically significant decline in happiness levels as the mental health disorder score increases. The mild group (6–8) was significantly happier than both the moderate group (13–17) and the severe group (18–28). Furthermore, the moderate group (9–12) was significantly happier than the severe group (18–28). Surprisingly, the healthy group (0–5) displayed significantly lower happiness levels than the mild group (6–8); perhaps this is due to the fact that the sample size of this group is the smallest compared to the others.

The study findings overall demonstrate that there is an inverse relationship between mental health severity and happiness levels. This suggests that individuals with higher scores on the mental health disorder scale were more likely to report lower levels of happiness. Other studies, including [17,18,19,20,21,22,23,24,25,26,27,28,29,30,31,32,33,34,35,36,37,38,39,40], also reported similar findings.

Limitations of the study include a small number of respondents from only one city in Saudi Arabia; therefore, this study can only be considered exploratory. Larger numbers of respondents, inhabiting different parts of Saudi Arabia are required for a more robust, experimental study. Additionally, although the instruments were translated into Arabic and previously validated, OHQ-29 [25,27,29] and GHQ [30,31,32], the culturally constructed nature of happiness itself [30] means that further qualitative research would be useful obtaining a more nuanced understanding what happiness means to these women in this time and place. Moreover, although the data was gathered during the COVID-19 pandemic, the study did not consider women who might be under exposure to the COVID-19, such as a frontline healthcare worker [41] or caregiver for a COVID-19 patient, which might have impacted on their happiness and mental health [42].

To summarize, this study contributes to the literature in examining Saudi women’s happiness and mental health to provide insights into increasing both, along with directions for future research. Happiness among women in general is important for individual, social, and economic wellbeing, as well as physical and mental health. Additionally, in the case of Saudi Arabia, both women and happiness are central to the success of the Vision 2030 reform initiative. Further research is required to examine the correlation between happiness and mental health among women in Saudi Arabia and assess their well-being. Attention needs to be paid to women’s sources of happiness and how they construct happiness across life domains in changing cultural times. Increasing rights and responsibilities are sure to bring additional sources of happiness, as well as stressors, and researchers need to keep track of this rapidly changing social group.

## 5. Conclusions and Recommendations

In conclusion, the current study investigated the relationship between happiness and mental health among Saudi women and found that the majority of these women are generally happy and mentally healthy. The study revealed that singles showed poorer mental health compared to married people, particularly with regard to anxiety, insomnia, and depression. This echoes previous research suggesting that married people have better mental health over the long term. The group of 15–20-year-old Saudi women showed the greatest depression score compared to the older groups and were also less happy than 21–30-year-old women. However, the study had limitations, including a small sample size from one city in Saudi Arabia and the need for further qualitative research to obtain a more nuanced understanding of what happiness means to these women in this time and place. Overall, this study suggests that various demographic factors can play a role in mental health and well-being and may need to be taken into consideration when developing interventions or providing support.

The study on the relationship between happiness and mental health among Saudi women suggests recommendations for improving participants’ mental health and happiness, including promoting mental health education, providing accessible mental health services, encouraging physical activity, implementing stress-management programs, and fostering social support and community connections. These recommendations aim to address cultural and social factors that impact women’s mental health in Saudi Arabia.

The study on mental health and happiness among Saudi women in Riyadh has several limitations that should be considered when interpreting its results. These limitations include using convenience sampling, which may not be representative of the entire population; excluding women with diagnosed mental health disorders or receiving treatment, potentially biasing the results towards healthier individuals; using self-reported measures of mental health and happiness, which may be subject to social desirability bias; relying on an online survey that may underrepresent certain groups in the sample; focusing only on sociodemographic characteristics and not including other factors that may impact mental health and happiness; and not conducting any follow-up assessments.

## Figures and Tables

**Table 1:** Demographic characteristics.

Variable	Number (%)
**Female (*n*.)**	308
**Age range (years)**	
15–20	84 (27.3%)
21–30	95 (30.8%)
31–40	81 (26.3%)
41–50	48 (15.6%)
**Marital status**	
Single	79 (25.6%)
Married	194 (63%)
Divorced	25 (8.1%)
Widow	10 (3.2%)
**Educational level**	
School	56 (18.2%)
Bachelor	203 (65.9)
Master	25 (8.1%)
PhD	24 (7.2%)
**Employment**	
Employed	179 (58.1%)
Unemployed	129 (41.9%)

**Table 2:** Oxford Happiness Questionnaire.

Variable	Mean ± *SD* or Number (%)
**Overall score**	4.22 ± 0.71
**Unhappy (1–2)**	2 (0.6%)
**Not particularly happy or unhappy (3–4)**	121 (39.3%)
**Happy (5–6)**	185 (60.1%)

**Table 3:** General Health Questionnaire.

Variable	Mean ± *SD*
**Overall score**	11.15 ± 6.60
**Somatic symptoms**	2.57 ± 1.87
**Anxiety and insomnia**	2.88 ± 2.69
**Social dysfunction**	4.01 ± 2.38
**Severe depression**	1.68 ± 2.20

**Table 4:** Happiness scores as related to mental health.

Factors	Score (Mean ± *SD*)	Correlation (*r*)					
		OHQ Overall	General Health Questionnaire				
			Overall	Somatic	Anxiety	Social	Depression
OHQ Overall	4.22 ± 0.71		−0.266 **	−0.284 **	−0.345 **	0.274 **	−0.393 **
Pleased with self	2.55 ± 1.530	0.110	0.088	0.068	0.128 *	−0.105	0.180 **
Life is rewarding	4.08 ± 1.489	0.427 **	−0.093	−0.095	−0.121 *	0.112	−0.147 *
Satisfied with life	4.48 ± 1.480	0.524 **	−0.222 **	−0.237 **	−0.234 **	0.203 **	−0.383 **
Look attractive	4.37 ± 1.707	0.611 **	−0.168 **	−0.173 **	−0.252 **	0.275 **	−0.293 **
Find beauty in things	4.95 ± 0.979	0.497 **	−0.161 **	−0.181 **	−0.181 **	0.160 **	−0.238 **
Can organise time	4.52 ± 0.911	0.226 **	−0.025	−0.034	−0.022	−0.015	−0.072
Mentally alert	4.57 ± 1.430	0.534 **	−0.079	−0.140 *	−0.178 **	0.258 **	−0.136 *
Happy memories	4.47 ± 1.726	0.594 **	−0.286 **	−0.299 **	−0.345 **	0.160 **	−0.370 **

* *p* < 0.05, ** *p* < 0.005.

## Data Availability

The data that support the findings of this study are available from the corresponding author upon reasonable request.
